# The Extent of Medication Errors During Hajj in the Kingdom of Saudi Arabia

**DOI:** 10.7759/cureus.41801

**Published:** 2023-07-13

**Authors:** Ibrahim A Alzaagi, Khalid M Alshahrani, Abdulrahman N Abudalli, Saud Surbaya, Rashid Alnajrani, Sheraz Ali

**Affiliations:** 1 Pharmaceutical Care Services, King Saud Medical City, Ministry of Health, Riyadh, SAU; 2 Primary Health Care Center, Mina Al-Wadi Hospital, Ministry of Health, Makkah, SAU; 3 General Directorate of Quality and Efficiency Medical Supply, Ministry of Health, Riyadh, SAU; 4 College of Health and Medicine, University of Tasmania, Hobart, AUS

**Keywords:** prescribing, hajj, pilgrims, outcome, medication error

## Abstract

Background

Medication errors are frequently identified in healthcare institutions and pose a risk to patients. The mass gathering during Hajj may expose the pilgrims to numerous health risks. No study has reported the extent of medication errors during Hajj in Saudi Arabia. We investigated the rate, nature, reporting, severity, and causes of medication errors in Hajj pilgrims.

Methodology

A retrospective analysis of medication errors reported by healthcare professionals was conducted from July 5, 2022, to July 15, 2022, at Mina Al Wadi Hospital, Saudi Arabia. This study included all medication error report forms collected during the Hajj season. The National Coordinating Council for Medication Error Reporting and Prevention Index was used to classify the severity of medication errors.

Results

There were reports of 43 medication errors in 3,210 prescriptions. The medication error incidence rate was 1.5% (43/3,210). The highest proportion of medication errors (83.72%, 95% confidence interval (CI) = 72.69-94.75) occurred during the prescribing phase, and 97% (95% CI = 93.16-100.0) of medication errors were classified as near misses. Wrong drugs (23.25%) and frequency (18.60%) were responsible for nearly half of the medication errors. Lack of drug information was the leading cause of reported medication errors (58.14%), followed by environmental, personnel, and workload issues (23.25%), and look-alike/sound-alike medication issues (18.60%).

Conclusions

This study found that the incidence of medication errors was consistent with the global standard, and many of them did not reach pilgrims and were preventable. This highlights the importance of targeted interventions. Incorrect medication was the common type of medication error, highlighting a crucial area for intervention and improvement. Lack of drug information was the primary underlying factor in the occurrence of medication errors. Pharmacists were more likely than other healthcare professionals to report medication errors, highlighting the importance of their involvement in improving medication safety among pilgrims. Future research needs to focus on examining the effectiveness of interventions (e.g., provision of education regarding medicines and medication review) in reducing medicine-related events during Hajj.

## Introduction

Medicines are crucial in healthcare and significantly improve patient outcomes when used rationally. Nonetheless, because medicines are frequently prescribed, they are also one of the most common sources of errors and adverse events in a healthcare setting [1]. Medication error is “any preventable event that may cause or lead to inappropriate medication use or patient harm while the medication is in the control of the healthcare professional, patient, or consumer. Such events may be related to professional practice, healthcare products, procedures, and systems, including prescribing, order communication, product labeling, packaging, and nomenclature, compounding, dispensing, distribution, administration, education, monitoring, and use” [2]. The National Coordinating Council for Medication Error Reporting and Prevention (NCC MERP) created an index to categorize preventable medication errors based on the severity of patient harm to help healthcare facilities and professionals consistently and systematically track medication errors [2]. In hospitalized patients, medication errors increase morbidity and mortality rates. Most preventable medicine-related injury, which can occur during any stage of the medication use process, is linked to medication errors. A recent review reported that the overall incidence of medication errors in healthcare settings in Saudi Arabia is 44% [3]. In addition, prescribing errors were the most common type of medication error in Saudi Arabian hospitals [3].

Every year millions of Muslims travel to Saudi Arabia to perform Hajj, one of the Islamic pillars and largest mass gatherings [4]. The government of Saudi Arabia provides the needed resources to serve the Hajj pilgrims via numerous ministries concerned (e.g., the Ministry of Hajj and Umrah and the Ministry of Health) [4]. The Ministry of Health operates 21 hospitals with a 7,000-bed capacity to provide healthcare to Hajj pilgrims [5]. Many older Hajj pilgrims visit Saudi Arabia with multiple comorbidities that are managed by medicines [6]. The mass gathering during Hajj may expose the pilgrims to numerous health risks [7]. There have been reports from healthcare providers of overcrowding in their clinics during Hajj, which may overstretch the locally deployed healthcare services for the Hajj pilgrimage [8]. Such a large number of medical complaints would require a similarly substantial volume of dispensed medicines, which are provided free of cost by the government during Hajj [9]. Evidence indicates that the average number of medicines prescribed per hospital outpatient visit during Hajj is 2.6 [10], which is over the World Health Organization’s reference value (1.6 to 1.8) for the average number of medicines prescribed per encounter [11]. The occurrence of medication errors is high in patients on multiple medicines [12]. No study has reported the extent of medication errors during Hajj in Saudi Arabia. Therefore, we investigated the rate, reporting, nature, severity, and causes of medication errors in Hajj pilgrims who visited a temporary healthcare facility in Makkah, Saudi Arabia. The results of this study may facilitate the development of prevention strategies and the implementation of awareness and educational workshops for healthcare professionals providing services during the Hajj season, thereby enhancing the medication use process and safety of Hajj pilgrims.

## Materials and methods

A retrospective analysis of medication errors reported by healthcare professionals was conducted from July 5, 2022, to July 15, 2022, at Mina Al Wadi Hospital, with a 160-bed capacity, in Mecca, Saudi Arabia [13]. This hospital provides healthcare services to diverse pilgrims during the Hajj season.

The process for anonymously reporting medication errors at Mina Al Wadi Hospital is intended for use by healthcare professionals. Similarly, all reports of medication errors were anonymous to overcome the problem of underreporting. Medication errors are reported voluntarily to the Medication Safety Unit during Hajj. The Medication Safety Unit is operated by two pharmacists who review and analyze medication errors, ensure healthcare professionals comply with medication safety policies and procedures, and enhance patient safety and the medication use process. Healthcare professionals (e.g., nurses, and pharmacists) reported medication errors to the Medication Safety Unit using a paper-based format known as the medication error reporting instrument. All report forms were evaluated by pharmacists from the Medication Safety Unit, who also reviewed the patient’s medical record and contacted the healthcare professional if clarification was needed. The following variables were included in the study: cause of errors, type of errors, error stage, error made or reported by, and error outcome. The NCC MERP Index for categorizing medication errors algorithm was used to determine the severity of the medication errors [2]. This non-invasive study was initiated after obtaining approval from the Institutional Review Board of King Saud Medical City (reference number: H1RI-19-Sep22-02).

Based on the temporary and unique nature of the Hajj pilgrimage, we estimated that our research would observe a moderate number of medication error occurrences, possibly between 30 and 50 cases. However, there is neither a previous study nor incidence data regarding medication errors during the Hajj season. This study included all medication error report forms collected during the Hajj season from July 5, 2022, to July 15, 2022. Data analysis was performed using SPSS version 27.0 (IBM Corp., Armonk, NY, USA). Descriptive statistics were used to calculate frequencies and percentages.

## Results

The data were collected over a period of 10 days from July 5, 2022, to July 15, 2022. We collected data on 3,210 prescriptions containing a total of 405 medications from four different departments, including the intensive care unit (ICU), emergency room (ER), medical ward, and outpatient department (OPD). A total of 43 medication errors were identified during the study period, with 23 occurring in males and 20 in females. The majority of patients with medication errors were between the ages of 40 and 60 years. The percentage of medication errors per prescription was 0.0133% (1.3 per 100 prescriptions). The departments with the highest incidence of medication errors were the OPD with 25 errors, followed by the ER with seven errors and the ICU with six errors, while the medical ward had five errors.

The most frequent types of medication errors were wrong drug and wrong frequency, which accounted for 23.25 (95% confidence interval (CI) = 10.62-35.88) and 18.60% (95% CI = 6.97-30.23) of errors, respectively (Table 1). Pharmacists identified and reported many medication errors (76.74%, 95% CI = 64.11-89.37) and made fewer errors (9.30%, 95% CI = 0.62-17.98) than physicians (81.39%, 95% CI = 69.76-93.02). Nurses reported only 23.25% of total medication errors. The main medication error causes were lack of drug information (58.14%), followed by environmental, staffing, and workload issues (23.25%), and look-alike/sound-alike medication problems (18.60%). The prescribing phase had the highest error rate (83.72%, 95% CI = 72.69-94.75), followed by the dispensing phase (9.30%, 95% CI = 0.62-17.98), and the administration phase (7.0%, 95% CI = 0.0-14.63) had the lowest error rate (Table 2). Of the 43 medication errors reported, 97.67% (95% CI = 93.16-100.0) corresponded to near misses, and only 2.32% of errors (95% CI = 0.0-6.82) reached the patients but did not cause injury (Table 3). 

**Table 1:** Frequencies and percentages of medication error types. CI = confidence interval

Type of error	n	Percent (95% CI)
Improper dose (over, under, or extra dose)	6	13.95 (3.59–24.31)
Wrong patient	3	7.00 (0.0–14.63)
Wrong drug	10	23.25 (10.62–35.88)
Wrong strength/concentration	4	9.30 (0.62–17.98)
Wrong frequency	8	18.60 (6.97–30.23)
Wrong route	2	4.65 (0.0–10.94)
Wrong dosage form	2	4.65 (0.0–10.94)
Wrong duration	6	13.95 (3.59–24.31)
Monitoring error, drug-drug interaction	1	2.32 (0.0–6.82)
Wrong time of administration	1	2.32 (0.0–6.82)
Total	43	100

**Table 2:** Stages, causes, reporting, and actions taken for resolution of medication errors. CI = confidence interval

		n	Percent (95% CI)
Stage(s) of error	Prescribing error	36	83.72 (72.69–94.75)
	Dispensing error	4	9.30 (0.62–17.98)
	Administration error	3	7.00 (0.0–14.63)
Error made by	MD/Physician	35	81.39 (69.76–93.02)
	Pharmacist	4	9.30 (0.62–17.98)
	Nurse	4	9.30 (0.62–17.98)
Error reported by	Pharmacist	33	76.74 (64.11–89.37)
	Nurse	10	23.25 (10.62–35.88)
Cause of error/Contributing factor	Clinical information missing (lab results or vital signs)	1	2.32 (0.0–6.82)
	Drug information missing	25	58.14 (43.39–72.89)
	Miscommunication of drug order (illegible, ambiguous, incomplete)	3	7.00 (0.0–14.63)
	Look-alike and sound-alike medication problem	8	18.60 (6.97–30.23)
	Environmental, staffing deficiency, and workload problem	10	23.25 (10.62–35.88)
	Lack of staff education and training problem	5	11.62 (2.04–21.20)
	Independent double-check system	3	7.00 (0.0–14.63)
Action taken for resolution (for pharmacists or nurses)	Call the physician for verification of an emergency order	19	44.18 (29.34–59.02)
	Clinical intervention	6	13.95 (3.59–24.31)
	Education and trained medical staff	3	7.00 (0.0–14.63)
	Send the pharmacist’s note to the physician for clarification	6	13.95(3.59–24.31)
	Change to the correct dose	1	2.32 (0.0–6.82)
	Drugs not dispensed to the patient	2	4.65 (0.0–10.94)
	Memo sent to the department	2	4.65 (0.0–10.94)
	Implementation of an independent double-check system	4	9.30 (0.62–17.98)
Interventions taken by physicians for resolution	Change to correct drug, dose, frequency, duration, and strength	40	93.02 (85.40–100.0)

**Table 3:** Percentage of medication errors classified by the degree of patient injury. Categories B-D were classified as no harm and categories E-I were classified as preventable adverse drug events. NCC MERP = National Coordinating Council for Medication Error Reporting and Prevention; CI = confidence interval

Classification	NCC MERP category	Definition	n	Percent (95% CI)
No error, no harm	A	Circumstances or occurrences with the potential to cause error	0	0.00 (0.0–0.0)
	B	An error occurred, but the patient was not affected (near miss)	42	97.67 (93.16–100.0)
	C	An error that reached the patient but did not result in patient injury	1	2.32 (0.0–6.82)
	D	An error occurred that reached the patient, necessitating monitoring to ensure that the patient was not harmed and/or intervention to prevent injury	0	0.00 (0.0–0.0)
Error, harm	E	An error occurred that may have caused or contributed to the patient’s temporary harm, necessitating intervention	0.00	0.00 (0.0–0.0)
	F	An error occurred that may have caused or contributed to the patient’s temporary injury, necessitating initial or extended hospitalization	0	0.00 (0.0–0.0)
	G	An error that may have caused or contributed to permanent patient injury	0	0.00 (0.0–0.0)
	H	An error occurred that necessitated a life-saving intervention	0	0.00 (0.0–0.0)
Error, death	I	An error that may have caused or contributed to the patient’s death	0	0.00 (0.0–0.0)
Unknown	U	Outcome is unknown	0.00	0.00 (0.0–0.0)
Total			43	100

## Discussion

The reporting of medication errors in temporary healthcare settings during Hajj is crucial for multiple reasons. By reporting medication errors, prompt corrective action can be taken to protect pilgrims from medicine-related injury. Medication errors during the Hajj pilgrimage can inform policy development and strategies to optimize medicine management during large-scale events such as Hajj.

This study examined the reporting of medication errors at a temporary healthcare facility in Saudi Arabia that operated only during Hajj. The incidence of medication errors was found to be 1.3 per 100 prescriptions, which is lower than a previous study conducted in the largest permanent tertiary care setting in Saudi Arabia, which reported an incidence of 1.5 per 100 prescriptions [14]. However, a recently published meta-analysis discovered significant variation in the reported rates of medication errors across Saudi Arabian hospitals [3]. Prior studies on medication errors were conducted in permanent tertiary care settings, which significantly differs from our study setting. The reported medication error rate in temporary healthcare settings is expected to be low during the Hajj season. This is because there are several obstacles to identifying and reporting medication errors. Temporary healthcare facilities during the Hajj season face unique obstacles, such as a transient population, constraints on resource allocation, infrastructure limitations, and time constraints. These factors can have a significant effect on the incidence and reporting of medication errors. Consequently, it may not be appropriate to explicitly compare our Hajj medication error data with studies conducted in permanent hospitals due to contextual differences. Instead, we should focus on identifying the specific difficulties and factors that contribute to medication errors in the temporary healthcare facility during the Hajj season. This strategy will yield valuable insights for enhancing patient safety and medication management in this unique setting.

Consistent with the results of a cohort study performed in Saudi Arabia, this research demonstrates that the vast majority of errors were near misses that could have been avoided [15]. Similar to previous studies, many medication errors occurred during the prescribing phase of medication use, with pharmacists identifying more than two-thirds of errors. Previous studies have discussed pharmacists’ roles in patient safety and noted that pharmacist interventions reduced medication errors by 11-89% during the medication use process [15-17]. The high rate of pharmacists reporting medication errors is a result of the pharmaceutical care services’ initiative to implement recognition awards for pharmacists. In our study setting, nurses reported medication errors at a low rate, while physicians did not report any errors. The Institute for Safe Medication Practices has identified a culture of appreciation for physicians and nurses as one of the best methods to increase the reporting of medication errors by other healthcare professionals [18]. Such efforts are necessary to address the widespread problem of underreported medication errors in Saudi Arabia [19]. The underreporting of medication errors in healthcare settings has been linked to the stigma associated with reporting them, thus it is important to foster a non-punitive work environment [20].

In our study setting, a lack of drug information was reported as the leading reason for reported medication errors. Improving patient safety and the medication error reporting system requires educating and raising awareness among healthcare professionals about the significance of reporting medication errors and implementing medication safety rules and procedures [21]. Consistent with earlier findings from studies conducted in Saudi Arabia and other settings, healthcare professionals frequently reported medication errors that occurred during the prescribing stage [15,22]. Early detection of prescribing errors permits prompt intervention and prevention of potential medicine-related injury, thus reducing healthcare costs [23].

This study revealed that more than 40% of medication errors involved incorrect frequency and incorrect drugs, and these errors have been regularly documented in other facilities [14,24]. By using barcode technology and computerized physician order entry, most types of medication errors can be prevented [25].

Several precautions were taken to prevent medication errors in the context of our study. These include the development and distribution of a look-alike/sound-alike medicines list using Tallman lettering, as well as the use of red auxiliary decals and physical separation for look-alike/sound-alike medicines. It was also suggested that pharmacists verify the diagnosis before dispensing medications, and nurses, pharmacists, and physicians receive educational lectures and awareness campaigns. In addition to the implementation of independent double-checks, educational sessions on the identification, reporting, and documentation of medication errors were provided to all physicians and chemists, and a blame-free environment was established. Medication management policies and procedures were developed and implemented. The restricted abbreviation, high-alert medication, and look-alike/sound-alike lists were updated and disseminated to all medical departments, and the prescribing privileges of medications were updated, approved, and distributed to physicians. We also employed pharmacists as medication safety officers to supervise the administration of medicines. These measures are intended to enhance medication safety and prevent medication errors in healthcare facilities.

Several factors may contribute to the underreporting of medication errors during the Hajj. These include a lack of comprehension regarding the reporting procedure and a lack of awareness among pilgrims regarding the need to report medication errors. Due to language barriers, it may be difficult for many pilgrims from non-Arabic-speaking countries to communicate with healthcare providers in Saudi Arabia. Due to the stigma surrounding medication errors and feelings of shame or humiliation, pilgrims may be discouraged from reporting incidents. In addition, limited access to healthcare facilities can inhibit the reporting of medication errors. It is crucial to resolve these obstacles to underreporting for increasing awareness regarding the importance of reporting and establishing safe and confidential reporting channels. Facilitating the identification and prevention of medication errors will increase patient safety during the Hajj pilgrimage.

Medication errors during the Hajj are a severe problem that can have a negative impact on pilgrims’ health and well-being. Medication errors may become more common during Hajj due to its particular difficulties, which include language barriers and cultural differences as well as the large number of pilgrims. To lower the risk of medication errors during Hajj, healthcare practitioners should combine a number of techniques, including standardizing medicine names and dosages, better communication, training for healthcare professionals, and the use of technology. Healthcare professionals should ensure that pilgrims receive the best care possible during this significant event by following these measures.

This research is the first of its kind that investigated the extent of medication errors during Hajj in a temporary healthcare setting. There were no previous data regarding medication errors during hajj. However, we compared our findings to all relevant literature. There are some limitations of this study. Because reporting medication errors at Mina Al Wadi Hospital is voluntary, the number of medication errors reported is likely to be underestimated. The medication safety personnel actively contacted healthcare personnel to encourage their participation in the reporting of medication errors. They also sent reminders to healthcare professionals to overcome the underreporting problem. Because this study was conducted in a single temporary healthcare setting during Hajj, its findings may not be representative of the entire group of Hajj pilgrims visiting Makkah. Future studies need to focus on enhancing the culture of reporting medication errors and obtaining a more comprehensive understanding of medicine safety in temporary healthcare settings during Hajj.

## Conclusions

This study found that the incidence of medication errors was consistent with the global standard, and many of them did not reach pilgrims and were preventable. This highlights the importance of targeted interventions. Wrong medication was the most common type of medication error, highlighting a crucial area for intervention and improvement. Lack of drug information was the primary underlying factor in the occurrence of medication errors. Pharmacists were more likely than other healthcare professionals to report medication errors, highlighting the importance of their involvement in improving medication safety among pilgrims. Future research needs to focus on examining the effectiveness of interventions (e.g., provision of education regarding medicines, and medication review) in reducing medicine-related events during Hajj. It is also crucial to examine determinants that could affect the reporting of medication errors during the Hajj season.
